# Chronic Stress Promotes Cancer Development

**DOI:** 10.3389/fonc.2020.01492

**Published:** 2020-08-19

**Authors:** Shirui Dai, Yongzhen Mo, Yumin Wang, Bo Xiang, Qianjin Liao, Ming Zhou, Xiaoling Li, Yong Li, Wei Xiong, Guiyuan Li, Can Guo, Zhaoyang Zeng

**Affiliations:** ^1^NHC Key Laboratory of Carcinogenesis and Hunan Key Laboratory of Translational Radiation Oncology, Hunan Cancer Hospital and the Affiliated Cancer Hospital of Xiangya School of Medicine, Central South University, Changsha, China; ^2^Key Laboratory of Carcinogenesis and Cancer Invasion of the Chinese Ministry of Education, Cancer Research Institute, Central South University, Changsha, China; ^3^Hunan Key Laboratory of Nonresolving Inflammation and Cancer, Disease Genome Research Center, The Third Xiangya Hospital, Central South University, Changsha, China; ^4^Department of Medicine, Dan L Duncan Comprehensive Cancer Center, Baylor College of Medicine, Houston, TX, United States

**Keywords:** stress, cancer, hormone, inflammation, immunity, hypothalamic-pituitary-adrenal axis, corticosteroids, catecholamines

## Abstract

Stress is an inevitable part of life. Chronic stress on account of reasons like adversity, depression, anxiety, or loneliness/social isolation can endanger human health. Recent studies have shown that chronic stress can induce tumorigenesis and promote cancer development. This review describes the latest progress of research on the molecular mechanisms by which chronic stress promotes cancer development. Primarily, chronic stress activates the classic neuroendocrine system [the hypothalamic-pituitary-adrenal (HPA) axis] and the sympathetic nervous system (SNS) and leads to a decline and dysfunction of the prefrontal cortex and the hippocampus under stress. Stress hormones produced during the activation of both the HPA axis and the SNS can promote tumorigenesis and cancer development through a variety of mechanisms. Chronic stress can also cause corresponding changes in the body's immune function and inflammatory response, which is significant because a long-term inflammatory response and the decline of the body's immune surveillance capabilities are implicated in tumorigenesis. Stress management is essential for both healthy people and cancer patients. Whether drugs that limit the signaling pathways downstream of the HPA axis or the SNS can suppress chronic stress-induced cancers or prolong patient survival deserves further study.

## Introduction

Humans have always experienced periods of excessive stress on account of global issues, such as poverty, war, and epidemics (1). Stress can be divided into acute stress and chronic stress. Acute stress usually exists in emergencies, such as fighting or escaping. Changes in the structure and function of certain molecules and tissues in the brain activate the emotional cognitive system, and we make decisions for stress-coping mechanisms (2). At the same time, the body temporarily produces catecholamines and corticosteroids to improve mobility and responsiveness. Therefore, acute stress is often beneficial to the body. However, chronic stress is heavily implicated in causing ill health, and today it is considered to encompass occupational stress as well as unusual adversities. Its potential negative effects include not only insomnia, gastrointestinal disorders (3, 4), anxiety, and depression (5, 6), but also an increased risk of cardiovascular disease, mental illness, and cancer (7).

Surveys have shown that approximately one million new cancer cases occur every year among young people aged 20–39 years (7), and they have been partly attributed to stress. The relationship between chronic stress and cancers has aroused increasingly widespread interest and concern in the medical community. Many scholars have performed research on the relationships between stress and cancers such as prostate (8–10), breast (8–12), gastric (13, 14), lung (15, 16), and skin cancer (17, 18), and have found evidence indicating that chronic stress can induce tumorigenesis and promote cancer development (Table 1).

**Table 1:** The mechanisms of chronic stress promoting cancer and corresponding targets and drugs.

**Cancer type**	**Mechanisms**	**The process of cancer**	**Targets**	**Drugs**
Breast cancer	Stress-catecholamines-ADRB2–LDHA-SLUG-stem-like properties	Proliferation Differentiation	LDHA	Vitamin C
	Stress-CRF-FAK phosphorylation, actin filament reorganization, prostaglandins	Invasion	CRF	Antalarmin
	Stress-glucocorticoids-glucocorticoid receptors-up-regulation of ROR1	Metastasis	ROR1	
	Stress restructures lymphatic networks within and around tumors	Metastasis	VEGFC	αVEGFC
	Stress activates COX-2/PGE2 system to affect the tumor microenvironment, including the induction of VEGFC production	Invasion Metastasis Angiogenesis	COX2	COX2i
	Stress-isoproterenol-ADRB2-upregulate the expression of CCL2 in pulmonary stromal cells and CCR2 in monocytes/macrophages	Metastasis	ADRB2	Propranolol
Gastric cancer	Stress-norepinephrine-ADRB2-adenosine 5′ monophosphate activated protein kinase unc 51 like autophagy activating kinase 1 (AMPK ULK1) pathway	Proliferation	ADRB2	β-blockers
	Stress-catecholamines-ADRB2-expression of VEGF, MMP-2, MMP-7, and MMP-9	Invasion Metastasis Angiogenesis	ADRB2	β-blockers (propranolol, ICI118,551)
Lung cancer	Stress-induced glucocorticoid surge and Tsc22d3 upregulation block the activation of type I IFN response in DCs and the IFN-γ+ T cells	Proliferation		
	Stress-norepinephrine-ADRB2-PKA-voltage-dependent calcium channels (VDCC)-activation of insulin-like growth factor (IGF)-1R	Metastasis	ADRB2 VDCC	CCBs (amlodipine, nifedipine)
Prostate cancer	Stress-catecholamines-ADRB2-alter of endothelial cell metabolism	Angiogenesis	ADRB2	β-blockers
	Stress-epinephrine-ADRB2-PKA-BAD	Apoptosis	ADRB2	β-blockers
			PKA	PKI
			BAD	BADS112A
Colon cancer	Stress activates COX-2/PGE2 system to affect the tumor microenvironment, including the induction of VEGFC production	Invasion Metastasis Angiogenesis	COX2	COX2i
	Stress-induced glucocorticoid surge and Tsc22d3 upregulation block the activation of type I IFN response in DCs and the IFN-γ+ T cells	Proliferation		
Skin cancer	Stress suppresses type 1 cytokines and protective T cells and increases the number of regulatory/inhibitory T cells	Proliferation		
Pancreatic cancer	Stress-catecholamines-ADRB2-expression of invasive genes	Invasion Metastasis	ADRB2	β-blockers

**Figure 1:**
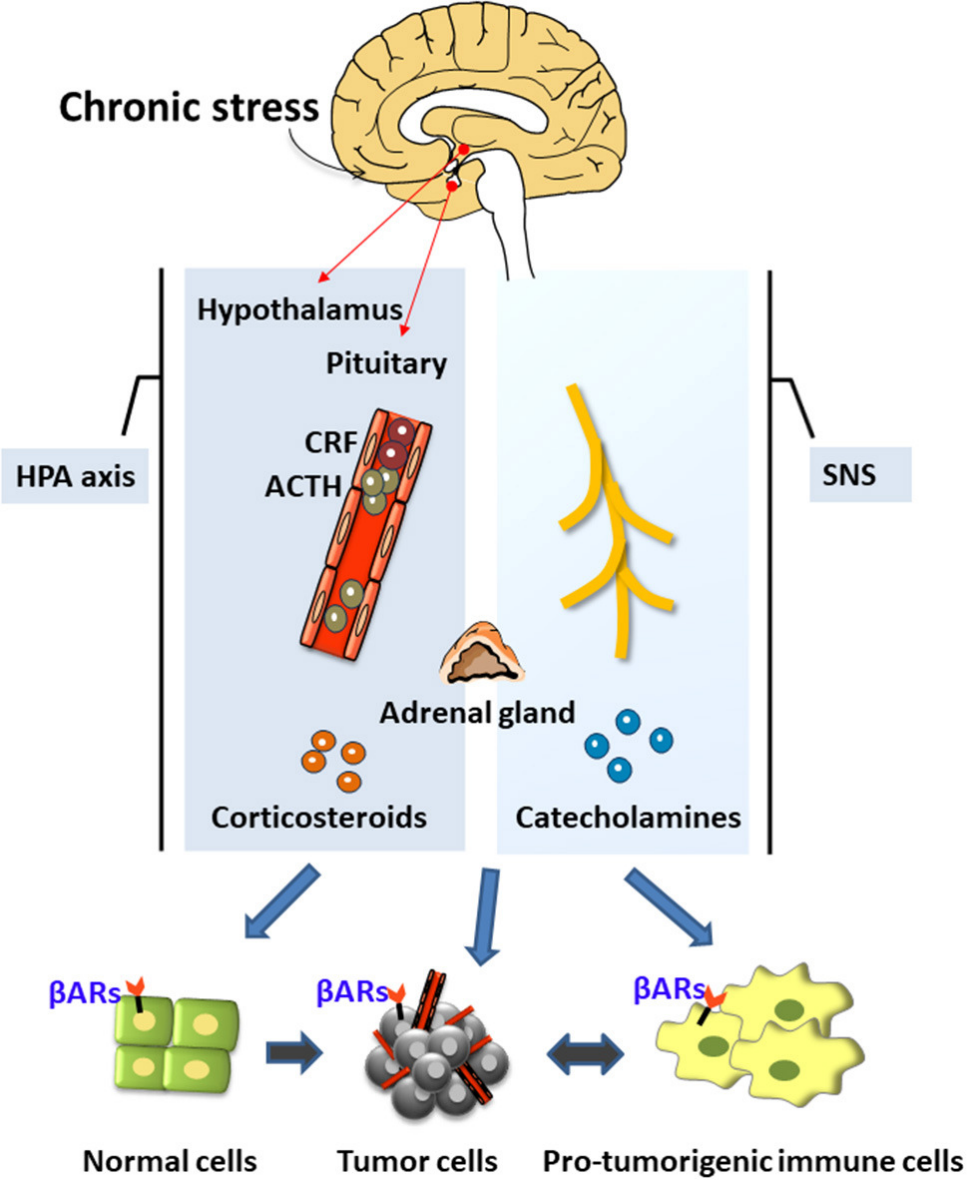
The neuroendocrine mechanisms of chronic stress. Chronic stress produces stress hormones during the activation of the neuroendocrine system (hypothalamus-pituitary-adrenal axis) and the sympathetic nervous system, which can promote tumor development and regulate the tumor microenvironment.

The neuroendocrine pathways are the most widely and comprehensively studied possible mediators of these associations. The neuroendocrine pathways constituting the hypothalamus-pituitary-adrenal (HPA) axis and the sympathetic nervous system (SNS) were the first systems shown to be closely related to stress [(1, 19); Figure 1]. Under chronic stress, the brain's nerve impulses can continuously activate the hypothalamus to produce the corticotropin-releasing factor (CRF). CRF is transported through the blood to the pituitary gland, thereby stimulating cells to release the adrenocorticotropic hormone (ACTH), which travels through the blood to the adrenal cortex and promotes the synthesis of corticosteroids. Chronic stress also activates the SNS, thereby stimulating the release of important neurotransmitters such as norepinephrine (NA) and adrenaline (Ad). NA and Ad are also hormones secreted by the adrenal medulla and known as catecholamines because they contain catechol and amine groups. The corticosteroids and catecholamines produced by the HPA and the SNS can cause a decline in the functions of the prefrontal cortex (20) and the hippocampus (21), and may enhance the activation of the SNS and the HPA by regulating the expression of glucocorticoid receptors (5, 22, 23).

Stress hormones promote the occurrence and development of cancers through various mechanisms such as by inducing DNA damage, increasing p53 degradation, and regulating the tumor microenvironment (Figure 1). Chronic stress can also activate the inflammatory response and the interaction between inflammatory cells and cancer cells to form the inflammatory tumor microenvironment, thereby promoting all stages of tumorigenesis (24). It can also enhance neuroinflammation, which further impairs the brain's cognitive processing of stress. This is a vicious circle. Chronic stress can also selectively suppress the type 1 helper T cells (Th1), suppress the cytotoxic T cells (CTL)-mediated cellular immunity and interferon production, and weaken immune surveillance and other processes, thereby increasing the risk of cancer invasion and metastasis and reducing the effectiveness of anti-tumor therapy (25, 26). In summary, chronic stress can promote tumorigenesis and oncogenesis through the production of stress hormones, the activation of inflammation, and the suppression of immunity. Therefore, the present review has focused on these three stress-mediated activities.

## Stress Hormones Promote Tumorigenesis and Cancer Development

Stress hormones are classified into classical corticosteroids and catecholamines, as well as the non-classical CRF and thyroid hormones.

### Classical Corticosteroids and Catecholamines

Corticosteroids include glucocorticoids and corticosterones. Elevated glucocorticoid levels increase the activity of the negative regulator murine double minute 2 (MDM2) through induction of the serum-and-glucocorticoid-regulated kinase (SGK1) and mediate the inhibition of p53 (27). P53 can initiate DNA repair, cell cycle arrest, aging, and apoptosis, which are related to the body's ability to inhibit tumor formation and respond to various types of cancer treatment (28). Therefore, the loss or impairment of the p53 function mediated by corticosteroids can considerably promote tumorigenesis. Obradović et al. (29) found that the increase in glucocorticoids during breast cancer progression was related to a lower survival rate. Increased hormone levels could lead to the activation of glucocorticoid receptors that were involved in the activation of multiple processes in metastasis and the up-regulation of kinase orphan receptor 1 (ROR1) at distant metastatic sites. Inhibition of ROR1 expression can reduce metastasis and prolong the survival rates of breast cancer patients.

Catecholamines can regulate the tumor microenvironment (30). In prostate cancer, catecholamines in the local sympathetic nerve fibers (NA) and circulating blood (Ad) can activate the β-adrenergic receptors (βARs) on endothelial cells, altering the cell metabolism, thereby inhibiting their oxidative phosphorylation, and inducing angiogenesis (31, 32). They can also activate the βARs in pancreatic cancer and stromal cells to increase the expression of invasive genes, thereby promoting the growth of primary tumors and the spread of tumor cells to adjacent tissues (33).

**Figure 2:**
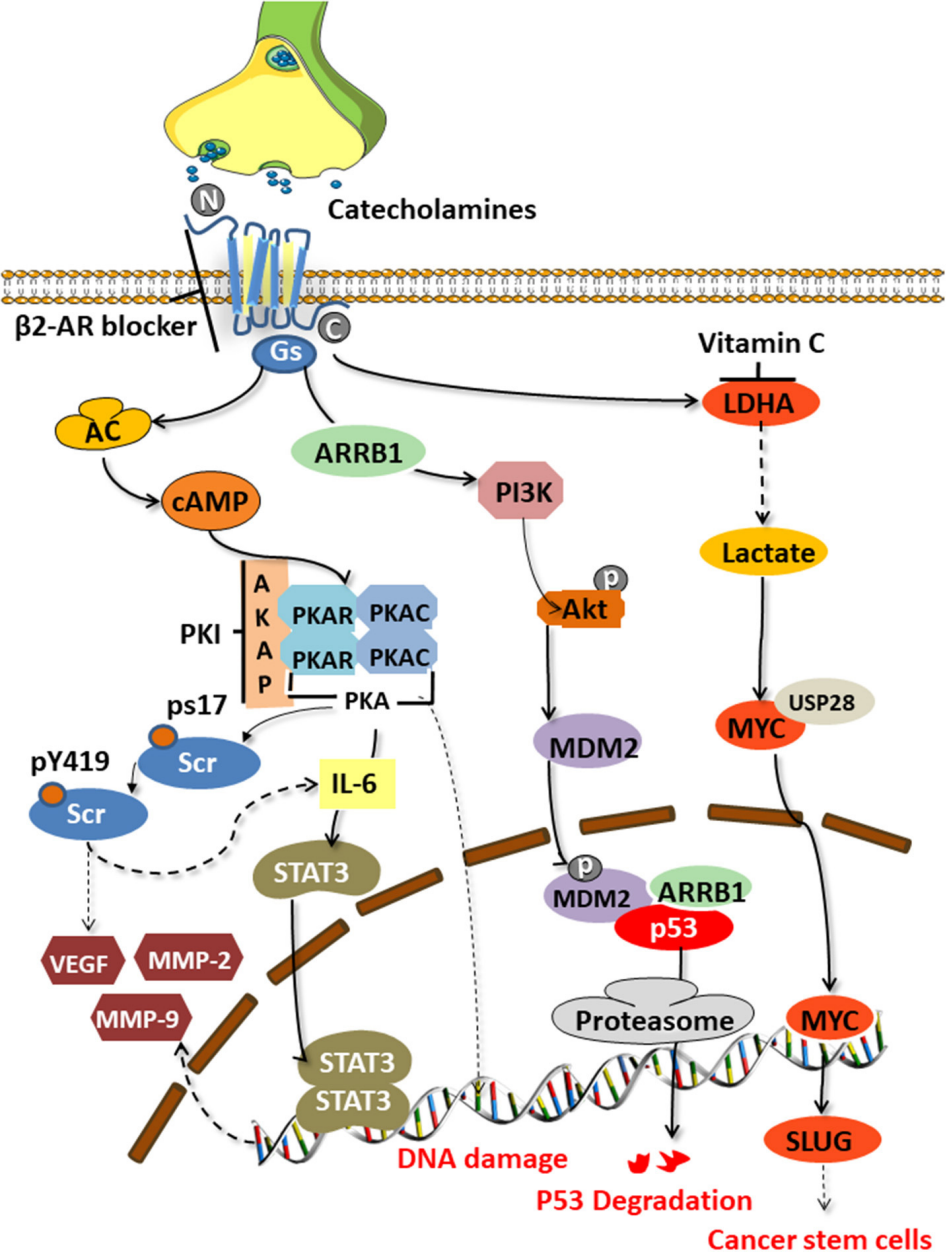
Signaling pathways activated by catecholamines. Stress-induced catecholamines activate the cAMP-PKA signaling pathway through β2 adrenergic receptors, thereby causing a series of downstream reactions. Src phosphorylation, DNA damage, p53 degradation, upregulation of VEGF and MMPs, and enhanced stem-like traits are key factors of tumorigenesis.

The tumor-promoting effect of catecholamines is mainly mediated by the β2 adrenergic receptor (encoded by ADRB2) activating the cAMP-protein kinase A (PKA) signaling pathway, which is the main mechanism to enhance tumor angiogenesis in the body and promote the growth of malignant cells [(34, 35); Figure 2]. The downstream mechanisms include mediating Src phosphorylation, DNA damage, p53 degradation, and the up-regulation of vascular endothelial growth factor (VEGF) and matrix metalloproteinases (MMP-2 and MMP-9). Catecholamines also mediate Src phosphorylation via βARs-cAMP-PKA, activate Ras-related protein 1 (Rap1), and inhibit extracellular signal-regulated kinases (ERKs), thereby enhancing tumor cell migration, invasion, and growth (36). Src is a non-receptor cytosolic tyrosine kinase that is involved in the VEGF and interleukin (IL-6) production in adipocytes and cancer cells, respectively (37).

Hara's team (38) clarified the specific mechanism of stress-mediated DNA damage. Catecholamines can stimulate the G-proteins (Gs)-PKA-mediated signaling pathways to trigger DNA damage and the β-arrestin (ARRB1)-mediated signaling pathways to trigger the Akt-mediated activation of MDM2, which promotes the binding and degradation of MDM2 and p53 by binding to M53. The synergy of these two pathways leads to the accumulation of DNA damage.

Angiogenesis is an important part of tumor development and metastasis. Studies have found significantly increased tumor blood vessel formation in stressed animals, and the mechanism maybe a βARs-cAMP-PKA-IL-6-dependent activation of signal transduction and activator of transcription (STAT3) (39). Activated STAT3 is transported to the nucleus and binds to specific DNA sites in the form of homo- or heterodimers, stimulating the transcription of the reactive genes *VEGF, MMP2*, and *MMP9* (34). VEGF plays a central role in the pathogenesis of various cancers as the best-known angiogenesis stimulator (40). When combined with its VEGF receptors on vascular endothelial cells, VEGF can promote tumor neovascularization. MMPs can degrade multiple protein components in the extracellular matrix, destroy the histological barrier of tumor cell invasion, and play a key role in tumor invasion and metastasis.

Cui et al. (8) found that chronic stress could induce adrenaline to activate lactate dehydrogenase A (LDHA) to produce lactic acid. The adjusted pH is conducive to the ubiquitin-specific protease 28 (USP28)-mediated ubiquitination and stabilization of MYC, and MYUG activates the SLUG promoter, which promotes the development of stem-like traits in breast cancer. Jang et al. (16) demonstrated that NA could induce the phosphorylation of the L-type voltage-dependent calcium channels (VDCC) via theβAR-PKA pathway. They further found that VDCC could trigger calcium mobilization, thereby inducing the activation of the insulin-like growth factor receptor (IGF-1R) and promoting cancer metastasis through the exocytosis of IGF2.

Motivated by studies of this mechanism, many researchers have tried to reverse the occurrence of the cancer-promoting effects by blocking the β2-AR and its downstream signaling pathway (Figure 2). Zhang et al. (41) found that by blocking the β2-ARs, a G1/S phase arrest and apoptosis could be induced in pancreatic cancer cells via the Ras/Akt/NFκB pathway. Hassan et al. (42) demonstrated an interaction between prostate cancers and the socio-psychological environment mediated by activating the Ad/ADRB2/PKA/BAD anti-apoptotic signaling pathway. They used the selective ADRB2 antagonist ICI118,551, or induced expression of the PKA inhibitor (PKI) or the BCL2-related death promoter BAD (BADS112A) with a mutated PKA phosphorylation site, to prevent the effects of stress in xenograft cancers. ADRB2 antagonists such as propranolol and ICI118,551 suppressed gastric cancer progression by inhibiting the ERK1/2-JNK-MAPK pathway and transcription factors, such as NF-κB, AP-1, and STAT3 (14). Cui et al. (8) conducted drug screening for LDHA and found that vitamin C could reverse the stem-like phenotype of breast cancer induced by chronic stress. Therefore, vitamin C may be a potential factor to combat stress-related breast cancer.

### CRF and Thyroid Hormone

CRF is widely present in the central nervous system, that has been detected in breast cancer tissues and cell lines, and affects breast cancer cell proliferation and invasion in an autocrine or paracrine manner (10). CRF can induce the phosphorylation of the focal adhesion kinase (FAK), induce Cox1 to produce prostaglandins, directly promote actin recombination and cell migration, and play a role in the TGFβ/SMAD2 and the Wnt-β-catenin signaling. The antagonist of CRF, antalarmin, inhibits neovascularization in 4T1 breast cancer cell lines *in vivo* (43).

Thyroid hormone is secreted by the thyroid gland, which is closely related to the body's metabolism, growth, and development, and is regulated by the hypothalamus-pituitary-thyroid axis. When people are emotional, their emotions stimulate the hypothalamus to release thyrotropin-releasing hormones and regulate the secretion of thyroid-stimulating hormones. This further affects the thyroid gland, and causes its gland cells to secrete a large amount of thyroid hormone. Frick et al. (44) found that under chronic stress, thyroid hormone levels and T cell lymphoid tissue hyperplasia response were reduced in animals and lymphoma growth was altered. The use of thyroxine replacement therapy can reverse the above process. Therefore, thyroid hormone may be a critical neuroendocrine regulator of tumor evolution, most likely through the regulation of T cell-mediated immunity.

## Chronic Stress Enhances Inflammation to Promote Tumor Development

Chronic stress and stress hormones can up-regulate the expression of stress-related pro-inflammatory genes in the circulating white blood cells, thereby increasing the release of pro-inflammatory cells and the production of pro-inflammatory cytokines, and can activate the aging-inflammatory response without the trigger of an exogenous inflammation, leading to the promotion of tumorigenesis and metastasis (45). Niraula et al. (46) found that high levels of corticosterone under repeated pressures of social failure promoted the release of the bone marrow-derived proinflammatory monocytes into the circulatory system and circulated in the brain, increasing neuroinflammation, and leading to a prolonged anxiety-like behavior. Inhibiting corticosterone overproduction through surgery (adrenalectomy) and drug therapy (metyrapone) can reduce these inflammatory responses to stress. Pretreatment of the socially disruptive-stressed mice with the β-adrenergic antagonist propranolol may prevent anxiety-like behaviors caused by social failure (47), reverse splenomegaly, and increase the levels of plasma inflammatory factors such as IL-6, tumor necrosis factor alpha (TNF-α), and the monocyte chemotactic protein 1 (MCP-1) (48). Moreover, research by Antoni's group shows that stress management in patients with early-stage breast cancer can reverse the up-regulation of the stress-related proinflammatory genes in white blood cells in the circulation (49).

**Figure 3:**
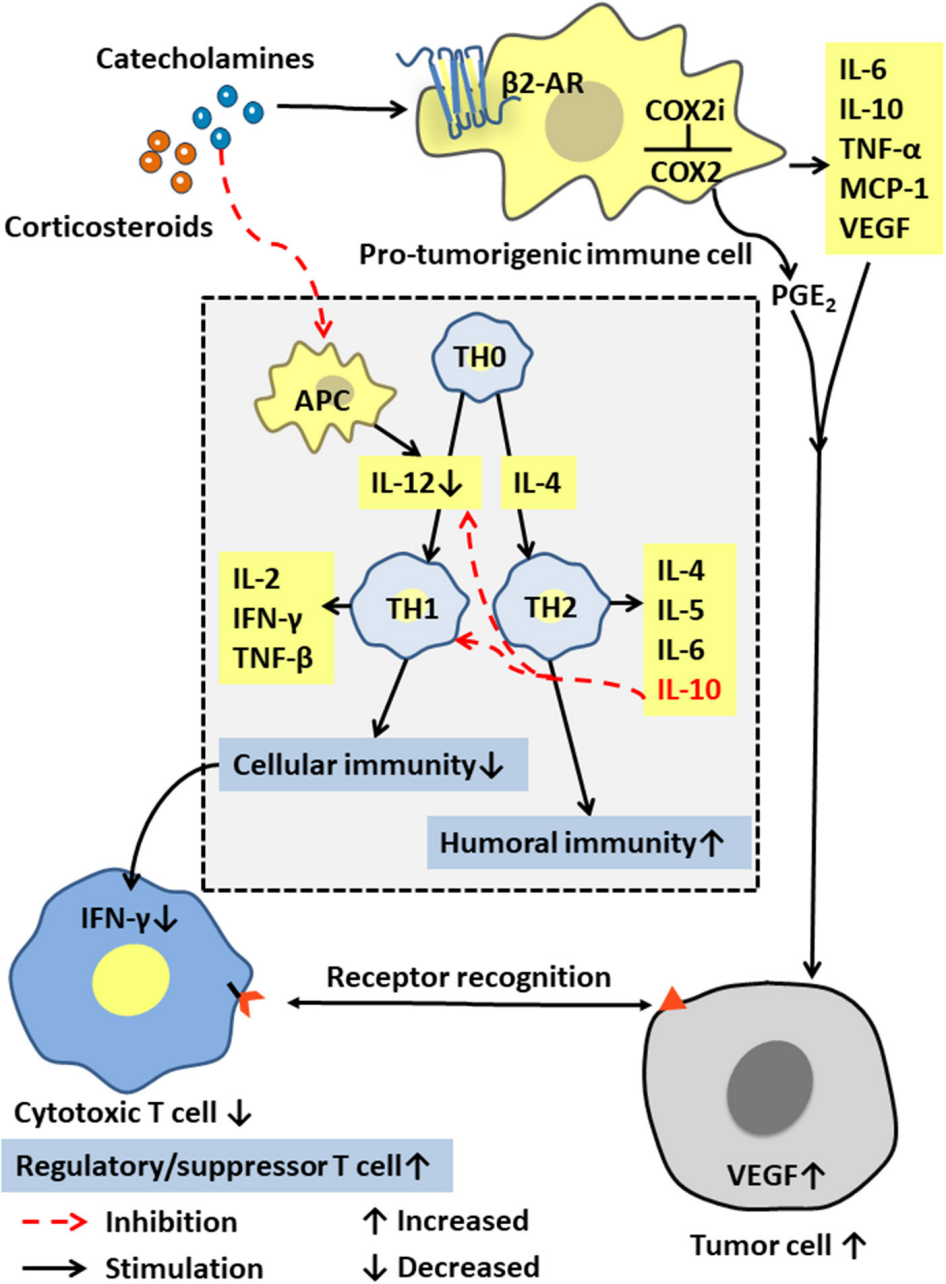
Effects of stress hormones on the immune system. Stress hormones stimulate pro-tumorigenic immune cells to produce IL-6, IL-10, and other cytokines, and activate the COX-2/PGE2 pathway to produce VEGF, which together affect the tumor microenvironment to suppress tumor immunity. The decrease in IL-12 and the increase in IL-10 lead to selective Th1 inhibition, thereby suppressing the CTL-mediated cellular immunity and interferon production.

Immune cells in the tumor microenvironment are called pro-tumorigenic immune cells and include the tumor-associated macrophages (TAMs), the dendritic cells (DCs), the myeloid-derived suppressor cells (MDSCs), and the tumor-infiltrating lymphocytes (TILs). These immune cells can produce cytokines such as IL-6, IL-10, TNF-α, and MCP-1, and are interconnected with tumor cells in an autocrine and paracrine manner. The pro-tumorigenic immune cells maintain a relative balance within a certain range of promoting tumor inflammation and anti-tumor immunity. After chronic stress breaks this balance through a long-term pro-inflammatory response, these cells and cytokines can act on all stages of tumor development, including initiation, promotion, malignant transformation, invasion, and metastasis through mutation, epigenetic modification, and regulation of the tumor microenvironment [(50, 51); Figure 3]. Moreover, activated inflammatory cells produce excess reactive oxygen species (ROS) to drive inflammation and mutagenesis through different pathways (52). The released cytokines can activate key transcription factors such as NF-κB (53–55) and STAT3 (56) in precancerous cells. By regulating the expression of many genes that can inhibit tumor cell death, promote tumor cell survival, and induce the production of chemokines, cytokines also attract more tumor-promoting immune cells to maintain a tumor-related inflammation.

## Chronic Stress Promotes Tumorigenesis and Cancer Development by Suppressing Immunity

As the core immune cells, thymus-dependent lymphocytes (T cells) provide a robust line of defense against infections and cancers. The cellular immunity mediated by them includes specific binding to their target cells for direct cell destruction or elimination and the release of cytokines to enhance and expand the immune effects. T cells can be roughly classified into the cytotoxic T cells, the helper T cells, and the regulatory/inhibitory T cells according to their functions. Based on different cytokine functions, the helper T cells are divided into Th1 or Th2 cells, which are differentiated from TH0 cells by their regulation by the cytokines IL-12 and IL-4. Th1 cells usually produce IFN-γ, TNF-β, and IL-2, and mediate cellular immune responses, such as inducing macrophage activation, mediating delayed hypersensitivity, and assisting CTL activation and proliferation. Th2 cells usually produces IL-4, IL-5, IL-6, and IL-10. Their main function is to enhance the humoral immune response and stimulate the B cells to produce antibodies. Notably, IL-12 and TNF-β promote a Th1 response and cellular immunity, while IL-10 inhibits IL-12 production and Th1 response and stimulates Th2 response and the humoral immunity (Figure 3).

Stress tests show that the plasma concentration of stress hormones is inversely proportional to the function of immune cells (57). Increased stress hormone production significantly reduces the activity of the antigen-presenting cells (APC), such as monocytes, macrophages, the dendritic cells, and the natural killer cells, that produce human IL-12 (58). Therefore, neuroendocrine mediators such as the stress-releasing glucocorticoids and epinephrine may selectively inhibit Th1, thereby inhibiting the CTL-mediated immune response and the production of interferon IFN-γ (26). Yang et al. (25) further confirmed that high levels of corticosteroids in plasma, and the up-regulated glucocorticoid-inducing factor Tsc22d3, blocked the activation of type I IFN response in DCs and IFN-γ+ T cells and reduced the efficacy of anti-tumor therapies in non-small cell lung cancer and colorectal cancer by suppressing the immune surveillance. Additionally, Saul et al. (17) found that chronic stress increased the sensitivity to the UV-induced squamous cell carcinoma by suppressing CTL and increasing the number of the regulatory/inhibitory T cells (Figure 3).

Stress hormones also induce cyclooxygenase COX-2 and a variety of COX-2-dependent inhibitors in human breast and colon cancer tissue cells, thereby activating the COX-2/PGE2 pathway (59). PGE2 is a biologically active lipid that can trigger inflammation and cancer. The activation of COX-2/PGE2 can affect the tumor microenvironment and inhibit tumor immunity through a variety of mechanisms, including inducing tumor cells to produce vascular endothelial growth factor C (VEGFC) and promoting the remodeling of lymphatic networks in and around tumors to provide a pathway for the tumor cells to escape the immune system [(60); Figure 3].

## Conclusions and Future Perspectives

Chronic stress can activate the HPA axis, and the SNS, and cause immune disorders and inflammatory responses. There is no doubt that this is harmful to the body. Excessive levels of stress hormones promote carcinogenesis by inducing DNA damage accumulation, increasing p53 degradation, and other, related pathways. Excessive stress hormones also prevent immune cells from effectively controlling cancer cells by increasing inflammation and suppressing immunity. Further, they can act on tumor and stromal cells in the tumor microenvironment to promote tumor growth, invasion, and metastasis. In addition to these pathways, emerging trends include investigation of the correlation between chronic stress and the microbiota-gut-brain axis (61–63), and its impact on intestinal diseases (64).

The effects of daily stress on the neuroendocrine and immune function of healthy human individuals, which may be modulated by the individual's personality, have been confirmed, for example, by Biondi et al. (65). Therefore, we need to actively manage stress (66, 67). A large amount of clinical evidence shows that supportive psychological therapy has a positive effect on anticancer treatment and prognosis of cancer patients (68, 69). In addition to increasing exercise (70) and psychological intervention (71–74) to regulate the patients' stress, we can also use drugs that limit the transmission of the HPA axis and the downstream signaling pathways of the SNS, such as the β-adrenergic receptor antagonists [(9); Figure 2], COX2 inhibitors (COX2i) [(59, 75); Figure 3], anti-VEGFC therapeutics (αVEGFC) or dopamine (76) (an inhibitory catecholamine). These drugs have been shown in animal experiments to not only significantly improve anxiety-like behavior (47, 48), and inhibit chronic tumor-promoting tumor growth, but also to block a stress-induced increase in angiogenesis and lymphatic metastasis (60). At the same time, we suggest that stress hormones should be used with caution, especially glucocorticoids (77) to treat patients with cancer and related complications. Finally, it is notable that β-blockers have been relatively widely used in clinical research (78, 79) and are often administered as adjuvants in cancer treatment in recent years [(80–84); Table 2], especially in breast cancer treatment. Though other related drugs have shown promise for treating cancer, there remains insufficient evidence for their clinical application.

**Table 2:** Clinical trials of stress-induced cancers in recent years.

**Cancer type**	**Study**	**No. of patients**	**Evaluation index**	**Drug use period**	**Drugs**
Breast cancer	Spera et al. (83)	1,144	Progression-free survival, overall response rate, and clinical benefit	During chemotherapy	β-blockers
	Shaashua et al. (84)	38	Epithelial-to-mesenchymal transition Transcription factors (GATA-1, GATA-2, EGR3, STAT-3) Tumor-infiltrating monocytes; B cells	Perioperative period	Propranolol Etodolac (COX2 inhibitor)
	Haldar et al. (82)	38	Pro-inflammatory cytokines (IL-6, CRP, and IFN-γ) Transcription factors (NF-κB, STAT3, ISRE) Proliferation marker Ki-67 PBMCs transcriptome	Perioperative period	Propranolol Etodolac
Lung cancer	Chaudhary et al. (81)	77	Pathological and imaging response, metastatic rate, and survival	During chemoradiotherapy	Propranolol
Ovarian cancer	Ramondetta et al. (80)	32	Anxiety, and depression Leukocyte expression of pro-inflammatory genes Serum IL-6, IL-8, IL-10	Before starting chemotherapy or surgery	Propranolol

## Author Contributions

SD, YM, and YW collected the related paper and finished the manuscript, tables, and figures. YL, WX, GL, CG, and ZZ gave constructive guidance and made critical revisions. BX, QL, MZ, and XL participated in the design of this review. All authors read and approved the final manuscript.

## Conflict of Interest

The authors declare that the research was conducted in the absence of any commercial or financial relationships that could be construed as a potential conflict of interest.

## Abbreviations

AR: adrenergic receptor
ACTH: adrenocorticotropic hormone
Ad: adrenaline
APC: antigen-presenting cell
ARRB1: β-arrestin
CRF: corticotropin releasing factor
DC: dendritic cell
FAK: focal adhesion kinase
HPA: hypothalamic-pituitary-adrenal
IGF-1R: insulin-like growth factor receptor
IL: interleukin
LDHA: lactate dehydrogenase A
MCP-1: monocyte chemotactic protein 1
MDM2: negative regulator murine double minute 2
MDSC: myeloid-derived suppressor cell
MMPs: matrix metalloproteinases
NA: norepinephrine
PKA: cAMP-protein kinase A
ROR1: receptor tyrosine kinase-like orphan receptor 1
ROS: reactive oxygen species
SGK1: serum-and-glucocorticoid-regulated kinase
SNS: sympathetic nervous system
STAT3: signal transduction and activator of transcription
T cell: thymus-dependent lymphocyte
TAM: tumor-associated macrophage
Th1: type 1 helper T cell
TIL: tumor-infiltrating lymphocyte
TNF-α: tumor necrosis factor alpha
VDCC: voltage-dependent calcium channel
VEGF: vascular endothelial growth factor.
